# How wheat *SIZ*es the day under pathogen attack

**DOI:** 10.1093/plcell/koag156

**Published:** 2026-06-01

**Authors:** Ju-Chen Chia

**Affiliations:** Assistant Features Editor, Plant Biology Section, The Plant Cell, American Society of Plant Biologists; School of Integrative Plant Science, Cornell University, Ithaca, NY, United States

Plant defense against pathogens relies on intricate signaling networks, among which the phytohormone salicylic acid (SA; 2-hydroxybenzoic acid) serves as a central regulator of immune responses. Upon microbial infection, plants rapidly elevate SA levels to activate systemic acquired resistance (SAR) (Vlot et al. 2021). Metabolic changes in response to environmental fluctuations can profoundly influence plant immune signaling, yet the mechanistic links between metabolism and disease defense remain incompletely understood. The branched-chain amino acids (BCAAs), including isoleucine (Ile), leucine (Leu), and valine (Val), are synthesized in chloroplasts and metabolized across multiple cellular compartments; they function not only as nutrients for bacterial and fungal pathogens, but also as signaling molecules that induce SA accumulation and subsequently promote SAR (Zeier 2013).

In wheat (*Triticum aestivum*), disruption of BCAA AMINOTRANSFERASE 1 (TaBCAT1), a key enzyme of BCAA metabolism, triggers the accumulation of BCAAs and SA, and induces *PATHOGENESIS-RELATED* (*PR*) genes. This decreases susceptibility to fungal pathogens, such as yellow rust (*Puccinia striiformis* f. sp. *tritici*; Pst) (Corredor-Moreno et al. 2021). However, the precise mechanism by which BCAAs activate this SA-dependent defense response has remained unclear.

Meanwhile, the SUMO E3 ligase SAP AND MIZ1 DOMAIN CONTAINING LIGASE1 (SIZ1) in *Arabidopsis thaliana* is known to function as a negative regulator of SA homeostasis. Arabidopsis loss-of-function *siz1* mutants constitutively accumulate SA, activate SAR, and display enhanced resistance to pathogen infection (Lee et al. 2007). A recent study further revealed an additional role for SIZ1 in promoting nuclear condensate-mediated immune activation (Jia et al. 2026). While SIZ1 and other components of the SUMOylation machinery are recognized as important regulators of biotic stress responses, little is known about how their transcription is regulated during pathogen attack.

**Swathy Puthanvila Surendrababu** and colleagues (Surendrababu et al. 2026) addressed these questions by investigating the role of *TaSIZ1* in the SA-mediated immune response of wheat and identifying a novel MYB transcription factor that regulates *TaSIZ1* expression in response to BCAA accumulation. The authors first showed that *TaSIZ1* functions similarly to *AtSIZ1* as a negative regulator of wheat immunity. They observed that *TaSIZ1* expression was repressed in the *TaBCAT1* disruption mutant, which accumulates high levels of BCAAs, thereby linking BCAA accumulation to *TaSIZ1* suppression. To identify upstream regulators of *TaSIZ1*, the authors compared the nuclear proteomes of wild-type wheat and the *TaBCAT1* disruption mutant. Among the transcription factors enriched in the mutant, 2 trihelix MYB/SANT proteins, 6b-INTERACTING PROTEIN-LIKE 1 (TaASIL1) and 6b-INTERACTING PROTEIN-LIKE 2 (TaASIL2), were predicted to recognize specific DNA binding motifs within the *TaSIZ1* promoter. Yeast one-hybrid assays further confirmed that TaASIL1 directly binds to the *TaSIZ1* promoter.

Next, the authors examined whether *TaASIL1* is required for BCAA-induced repression of *TaSIZ1* and the subsequent activation of SA signaling. Silencing *TaASIL1* in the *TaBCAT1* disruption mutant led to significantly increased *TaSIZ1* expression while reducing *PR1* and *PR3* expression. In contrast, silencing *TaASIL1* in the wild type had little effect on *TaSIZ1* expression and caused only a minor upregulation of *PR3*. Consistent with a BCAA-dependent regulatory mechanism, overexpression of *TaASIL1* reduced *TaSIZ1* transcripts in the *TaBCAT1* mutant but increased *TaSIZ1* expression in wild-type plants. These genetic data suggest that TaASIL1-mediated *TaSIZ1* suppression depends on elevated BCAA levels.

To further clarify the link between BCAAs, TaASIL1/TaASIL2 function, and the transcriptional regulation of *TaSIZ1*, the authors characterized Kronos TILLING mutants carrying disrupted *TaASIL1* (TaASIL1-B^W31*^) or *TaASIL2* (TaASIL2-B^G191A^) alleles. In the TaASIL1-B^W31*^ mutant, Leu treatment failed to repress *TaSIZ1* expression or induce SA accumulation and only partially induced the expression of *PR* genes. Consequently, pathogen biomass was significantly higher in TaASIL1-B^W31*^ plants than in the wild-type controls following infection. These results suggest that TaASIL1 is required for the BCAA-induced immune response. In parallel, although TaASIL2 did not directly bind to the *TaSIZ1* promoter, the TaASIL2-B^G191A^ mutant showed elevated *TaASIL1* expression. The TaASIL2-B^G191A^ plants also accumulated higher levels of BCAAs and exhibited reduced pathogen biomass compared with the wild-type plants. These results indicate that TaASIL2 negatively influences BCAA accumulation and the TaASIL1-related pathway.

Together, these findings provide new insights into BCAA-mediated disease resistance in wheat by identifying the BCAA-TaASIL1-TaSIZ1 pathway in SA signaling (Fig. 1). These newly identified components represent promising targets or molecular markers for breeding crops with enhanced resistance to devastating rust fungi. This study further highlights how trihelix MYB/SANT transcription factors may function as key mediators linking metabolic status to SUMOylation-associated stress responses in plants.

**Figure 1:**
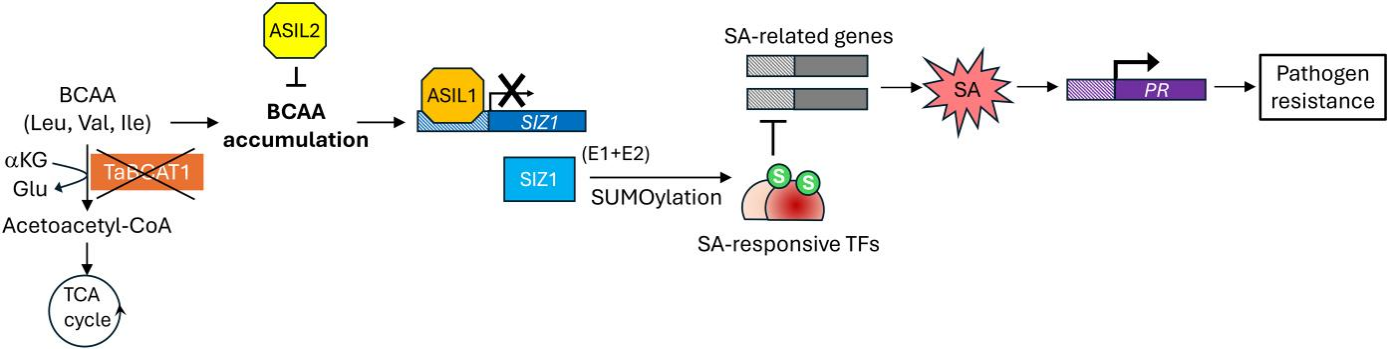
Model illustrating the role of TaASIL1 and TaSIZ1 in linking elevated BCAA levels to SA-mediated immunity. Accumulation of BCAAs following disruption of TaBCAT1 promotes TaASIL1-mediated repression of *TaSIZ1* transcription, which, in turn, enhances free salicylic acid (SA) accumulation and pathogenesis-related (*PR*) gene expression, thereby increasing resistance to fungal pathogens. TaASIL2 negatively influences BCAA accumulation and thus also contributes to the regulation of the TaASIL1-TaSIZ1 pathway. Modified from Surendrababu et al. (2026), Figure 8.

## Recent related articles in *The Plant Cell:*

Coleman et al. (2026) showed that HSFA1 transcription factors activate wound signaling and cellular reprogramming in Arabidopsis, while SIZ1-mediated SUMOylation attenuates HSFA1 activity and negatively regulates the HSFA1-dependent transcriptional cascade.

Huang et al. (2025) reported that the Arabidopsis SUMO E3 ligase SIZ1 stabilizes WRI1 through SUMOylation to maintain seed filling and fatty acid biosynthesis under prolonged high-temperature stress.

Xiao et al. (2025) revealed that salicylic acid signaling plays a central role in cold-induced proline accumulation and cold tolerance in *Citrus*. The authors showed that CtrTGA2, a TGACG sequence-specific binding protein, coordinates SA biosynthesis and proline accumulation through transcriptional activation of *CtrICS1*, which encodes isochorismate synthase, and *CtrP5CS1,* which encodes pyrroline-5-carboxylate synthetase.

## Data Availability

No new data were generated or analysed in support of this research.
